# Low-dose energetic protons induce adaptive and bystander effects that protect human cells against DNA damage caused by a subsequent exposure to energetic iron ions

**DOI:** 10.1093/jrr/rrv005

**Published:** 2015-03-23

**Authors:** Manuela Buonanno, Sonia M. De Toledo, Roger W. Howell, Edouard I. Azzam

**Affiliations:** 1 Department of Radiology, New Jersey Medical School Cancer Center, Rutgers University, 205 South Orange Avenue, Newark, NJ 07103, USA; 2 Present address: Center for Radiological Research, Columbia University Medical Center, 630 West 168th Street, New York, NY 10032, USA

**Keywords:** space radiation protection, protons, high atomic number and high energy (HZE) particles, adaptive protection, bystander effect

## Abstract

During interplanetary missions, astronauts are exposed to mixed types of ionizing radiation. The low ‘flux’ of the high atomic number and high energy (HZE) radiations relative to the higher ‘flux’ of low linear energy transfer (LET) protons makes it highly probable that for any given cell in the body, proton events will precede any HZE event. Whereas progress has been made in our understanding of the biological effects of low-LET protons and high-LET HZE particles, the interplay between the biochemical processes modulated by these radiations is unclear. Here we show that exposure of normal human fibroblasts to a low mean absorbed dose of 20 cGy of 0.05 or 1-GeV protons (LET ∼ 1.25 or 0.2 keV/μm, respectively) protects the irradiated cells (*P* < 0.0001) against chromosomal damage induced by a subsequent exposure to a mean absorbed dose of 50 cGy from 1 GeV/u iron ions (LET ∼ 151 keV/μm). Surprisingly, unirradiated (i.e. bystander) cells with which the proton-irradiated cells were co-cultured were also significantly protected from the DNA-damaging effects of the challenge dose. The mitigating effect persisted for at least 24 h. These results highlight the interactions of biological effects due to direct cellular traversal by radiation with those due to bystander effects in cell populations exposed to mixed radiation fields. They show that protective adaptive responses can spread from cells targeted by low-LET space radiation to bystander cells in their vicinity. The findings are relevant to understanding the health hazards of space travel.

## INTRODUCTION

During prolonged space missions, astronauts can be exposed to ionizing radiation (IR) from a variety of sources, including galactic cosmic rays and solar protons. Galactic cosmic rays consist mainly of protons, but a small fraction of them are high atomic number (Z) and high energy (E) (HZE) particles [1]. The linear energy transfer (LET) of protons is low, whereas HZE particles have intermediate and high LET. Although of low abundance in space, HZE particles can generate significant health effects because of their high radiotoxicity per unit absorbed dose compared with the more abundant protons [2, 3]. Astronauts may be also exposed to secondary radiations such as neutrons and recoil nuclei that result from the interaction of incident particles with nuclei of atoms of spacecraft material or their bodies [4, 5]. During a mission to Mars (∼1000 days), every cell nucleus in an astronaut's body is likely to be hit by a proton or a secondary electron every few days, and by an HZE particle about once a month [6]. Therefore, on average during a Mars mission, a given cell is traversed by ∼10 protons before it is traversed by an HZE particle. These diverse types of IR may impart biological effects mediated by hitherto unknown mechanisms. For example, an extensive cross-talk may occur among various molecular and biochemical events modulated by these radiations, which may determine the severity of the health effects. Thus, one of the goals of this study was to assess DNA damage in normal human cells exposed to protons followed at a subsequent time by exposure to energetic HZE particles. In addition, the spread of signaling events from low-dose proton-irradiated cells to non-irradiated cells in their vicinity (i.e. bystanders), and the ensuing response of the latter cells to a challenge by HZE particles is also examined.

Currently, for the purposes of radiation protection, the deleterious effects of IR are assumed to have a linear dose response with no threshold (discussed in e.g. [7, 8]). The effects of sequential doses are presumed to be additive. One consequence of this assumption is the notion that exposure to any dose of radiation, however small, increases the risk of detrimental radiation-induced health effects. Observations of adaptive and bystander effects challenge this assumption [9]. Adaptive responses and bystander effects of both protective and detrimental nature have been widely observed in cell cultures exposed to IR, and this is leading to a paradigm shift in our understanding of the IR target [10–13]. Significant evidence from different laboratories indicates that extranuclear and extracellular events can contribute to important biological changes in both the directly irradiated cells and the surrounding bystander cells [13–16]. While adaptive responses are thought to mitigate the harmful effects of IR, bystander effects have been generally suggested as amplifying the consequences of irradiation.

Adaptive responses encompassing DNA repair and antioxidation reactions have been reported to have been induced following exposures to low doses of low-LET radiations, in particular of γ rays delivered at low dose rate [17–22]. The protective mechanisms defend against the damaging effects of a subsequent exposure to IR [23–26] and against spontaneous damage due to normal physiological processes [17, 27, 28]. Interestingly, genes responsible for both generating and scavenging reactive oxygen species (ROS) were upregulated in the livers of mice that were flown on a 13-day space shuttle mission (STS-118) [29]. However, more recent reports have indicated that exposure to a low fluence of low-LET protons propagated signaling factors that induced DNA damage in bystander cells [30]. In fact, the lack of clear knowledge about non-targeted responses of space radiations [31–33] has been singled out by the US National Academies [34] as one of the most important factors limiting the prediction of radiation health risks associated with deep space exploration. Whereas several studies have shown that HZE particles are strong inducers of harmful bystander effects [33, 35–38], studies of proton-induced adaptive and bystander responses are only emerging.

## MATERIALS AND METHODS

### Cells

Apparently normal human diploid skin fibroblasts (AG1522) were obtained from the Genetic Cell Repository at the Coriell Institute for Medical Research (Camden, NJ). Cells at passages 10–12 were grown in Eagle's Minimum Essential Medium (CellGro) supplemented with 12.5% heat-inactivated (56°C, 30 min) fetal calf serum (FCS), 200 mM L-alanyl-L-glutamine, 100 U/ml penicillin and 100 μg/ml streptomycin (Sigma). The cells were routinely maintained at 37°C in a humidified incubator with 5% CO_2_ in air. For experiments, cells were seeded at a density that allowed them to reach the confluent state within 5 days. They were then fed twice on alternate days, and experiments were initiated 24–48 h after the last feeding. Under these conditions, 90–98% of the cells were in G_0_/G_1_ phase of the cell cycle as determined by [^3^H]-thymidine uptake and/or flow cytometry [39]. Synchronization of the cells in G_0_/G_1_ phase by density inhibition eliminates complications in interpretation of the results (that arise from changes in the cellular response to IR at different phases of the cell cycle) [40].

### Irradiation

Irradiations were conducted at the NASA Space Radiation Laboratory (NSRL) located at the Brookhaven National Laboratory (Upton, NY). Description of the facility and radiation beam information can be found at www.bnl.gov/nsrl/userguide/, 10 March 2015, date last accessed. Confluent cell cultures were irradiated with 20 cGy from 0.05 or 1-GeV protons (^1^H^+^) (LET ∼ 1.25 and 0.2 keV/μm in water, respectively) at a dose rate of 0. 1 Gy/min. After 6–24 h, they were exposed to a mean absorbed dose of 50 cGy from 1 GeV/u iron ions (^56^Fe^26+^) (LET ∼ 151 keV/μm in water) at a dose rate of 0.5 Gy/min. The culture flasks were positioned orthogonal to the beam in the plateau region of the Bragg curve, but were not stacked (profiles of the Bragg curves can be accessed at http://www.bnl.gov/nsrl/userguide/bragg-curves-and-peaks.php, 10 March 2015, date last accessed). The flasks were filled to capacity with pH- and temperature-equilibrated growth medium 3–6 h before the radiation exposure. This ensured that, during the irradiation, deviation from 37°C was attenuated and the cells were immersed in medium, thus alleviating the changes in osmolarity and partial oxygen tension that can greatly affect the cellular radiation response [41, 42]. The irradiating particles impacted orthogonally, first the bottom of the plastic growth surface of the culture vessel, followed by the adherent cells and then the growth medium. Control cells were sham-treated and handled in parallel with the test cultures. Dosimetry for the experiments was performed by the NSRL physics staff.

### Cell culture strategy for bystander studies

To examine radiation-induced bystander effects, a layered tissue culture system that allows isolation of pure bystander cells from contiguous irradiated cells was used. Briefly, AG1522 fibroblasts destined to be bystanders were seeded onto inverted Transwell® inserts (i.e. on the underside of the insert) with 3-μm pores. Following attachment, the inserts were inverted and placed into the wells of plates and cultured to confluency as described above. Irradiated cells, derived from confluent cultures maintained in flasks, were harvested within 10 min following exposure to 20 cGy of energetic protons. The harvested cells were then seeded at confluent density on the top side of the insert, with bystander cells growing upon its underside (see scheme in Fig. 2A). In the 2-h period after plating, irradiated cells adhere and form functional junction channels with bystander cells, as was assessed by the transfer of Calcein AM dye [43, 44]. Irradiated and bystander cells may also communicate with each other through diffusible factors transferred across the pores of the membrane. The irradiated cells and contiguous bystanders were left in co-culture for a total of 5 h. Subsequently, the bystander cells were harvested and plated in tissue culture flasks at confluent density. Six to 24 h after plating, the bystander cells were exposed to a mean absorbed dose of 50 cGy of 1 GeV/u iron ions. In this experimental system, bystander cells that were co-cultured with the irradiated cells were neither traversed by the priming protons nor their δ rays or secondary fragmentation products, and they would not be affected by activated growth medium [36].

### Micronucleus formation

Radiation-induced DNA damage was assessed by measuring the frequency of micronuclei by the cytokinesis-block technique [45]. Briefly, 2 × 10^4^ cells were seeded in chamber flaskettes (Nalge Nunc International) in the presence of 2 μg/ml cytochalasin B (Sigma). At this concentration, cytochalasin B was not toxic to the cells, as assessed by colony formation. After 72 h incubation, the cells were rinsed in phosphate-buffered saline (PBS), fixed in ethanol, stained with Hoechst 33342 (1 μg/ml in PBS), and viewed with a fluorescence microscope. At least 1000 cells per treatment were examined, and only micronuclei in binucleated cells were considered for analysis. The fraction of micronucleated cells and the distribution of micronuclei per binucleated cell were evaluated. The distribution of micronuclei is presented as the percentage of binucleated cells exhibiting up to five micronuclei fragments, relative to the total number of cells examined. The percentage of binucleated cells in the cell population was ∼40%. The results of separate experiments were averaged, and Poisson statistics were used to calculate the standard error associated with the percentage of micronucleated cells in the total number of binucleated cells. Comparisons between treatment groups and respective controls were performed using the Pearson's χ^2^ test. A *P* value of ≤ 0.05 between groups was considered significant.

## RESULTS AND DISCUSSION

### Low doses of energetic protons confer protection against subsequent exposure to HZE particles

**Fig. 1.**
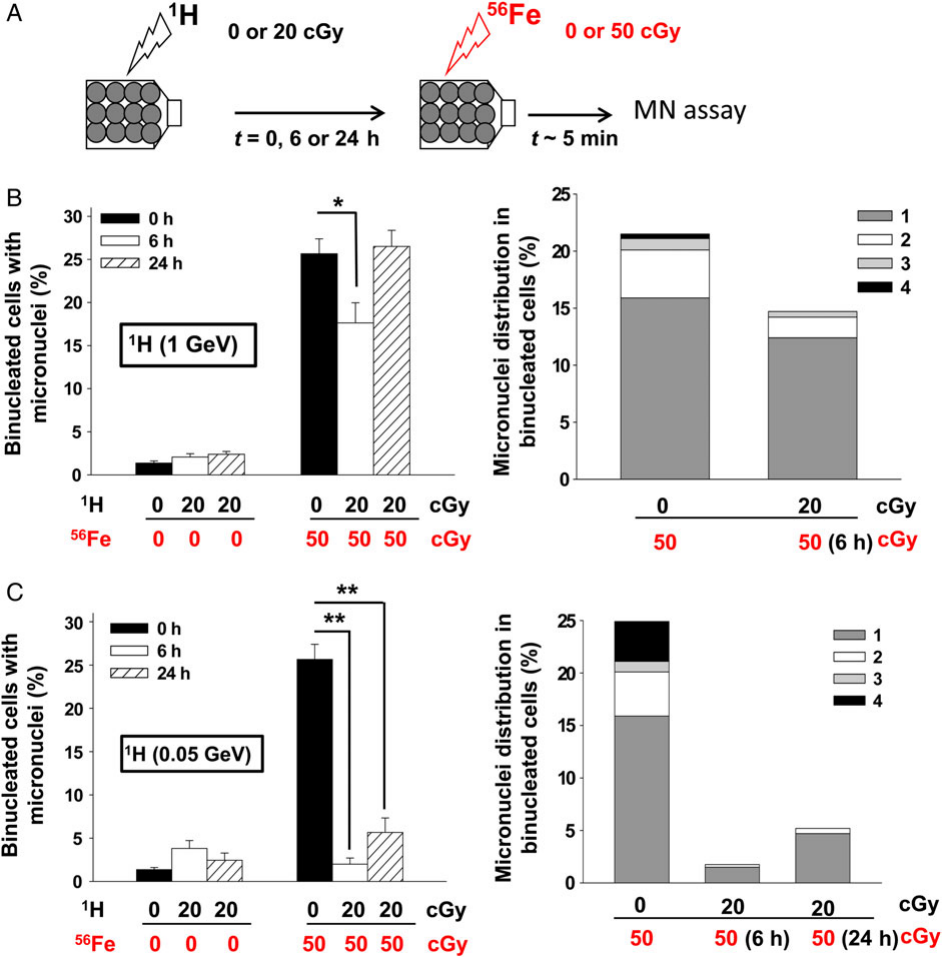
Proton-induced adaptive response in normal human cells. (**A**) Experiment schematic: Confluent normal human fibroblast cultures were first exposed to a priming radiation dose from energetic low-LET protons, followed 0, 6 or 24 h later by a challenge dose of high-LET iron ions. (**B**) Micronucleus formation (left panel) and micronuclei distribution (right panel) in AG1522 cells pre-irradiated with 20 cGy from 1-GeV protons (^1^H) and challenged 0, 6 or 24 h later with 50 cGy of 1-GeV/u iron ions (^56^Fe). (**C**) Micronucleus formation (left panel) and micronuclei distribution (right panel) in AG1522 cells pre-irradiated with 20 cGy from 0.05-GeV protons (^1^H) and challenged 0, 6 or 24 h later with 50 cGy of 1-GeV/u iron ions (^56^Fe). The data indicate that pre-exposure to a low dose of low-LET protons protects against DNA damage from a subsequent challenge from high-LET iron ions. *P < 0.05, **P < 0.0001.

We evaluated the DNA damaging effects of sequential exposures to low absorbed doses of low- and high-LET space radiations. Confluent AG1522 normal human fibroblast cultures were first exposed to a priming dose of 20 cGy from 0.05 or 1-GeV protons (LET ∼ 1.25 and 0.2 keV/μm, respectively), which results in uniform irradiation of the cell populations. Following 6- or 24-h incubation at 37°C, the cell cultures were challenged with a mean absorbed dose of 50 cGy from 1 GeV/u iron ions (LET ∼ 151 keV/μm), which targets essentially all the exposed cells with one or more HZE tracks [37] (see scheme in Fig. 1A). Within 5–10 min following exposure to the challenge dose, the cells were subcultured and assayed for micronucleus formation. When the cells were primed with 1-GeV protons and challenged 6 h later with energetic iron ions, the percentage of micronucleated cells was reduced from 28.9 ± 1.7% (only Fe ions) to 17.6 ± 2.3% (protons + Fe ions) (*n* = 3, *P* < 0.005) (Fig. 1B, left panel). Priming the cells before exposing them 6 h later to a challenge dose from iron ions also reduced the number of micronuclei per binucleated cell (Fig. 1B, right panel). The mitigating effect was transient and disappeared by 24 h (Fig. 1B, left panel).

A similar mitigating, but more persistent, effect was observed when the cells were primed with 20 cGy of 0.05-GeV protons (Fig. 1C, left panel). Here, the protective effect was evident for at least 24 h, with the percentage of micronucleated cells decreasing from 28.9 ± 1.7% (only Fe ions) to 2.0 ± 0.7% (protons + Fe ions) (*n* = 2, *P* < 0.0001) when the challenge dose was delivered 6 h after the priming dose, and to 5.7 ± 1.7% when it was delivered 24 h later (*n* = 3, *P* < 0.0001). The mitigating effects can be observed also in terms of reduction in the number of micronuclei per binucleated cell (Fig. 1C, right panel).

The above results are in contrast with previous studies in which it was shown that pre-irradiation with protons failed to protect primary human fibroblasts or epithelial cells against the damage induced by HZE particles delivered several hours or days after a proton priming dose [46–50]. The qualitatively different responses observed in these studies may be due to the fact that radiation-induced adaptive responses depend upon many parameters, including cell type, magnitude of the priming dose, energy and LET of the proton priming dose, dose rate, redox environment, stage of the cell cycle, and in particular expression time (i.e. the time separating the priming and challenge exposures (reviewed in [19, 51]).

### Proton-radiation-induced protective effects are propagated to neighboring bystander cells

**Fig. 2.**
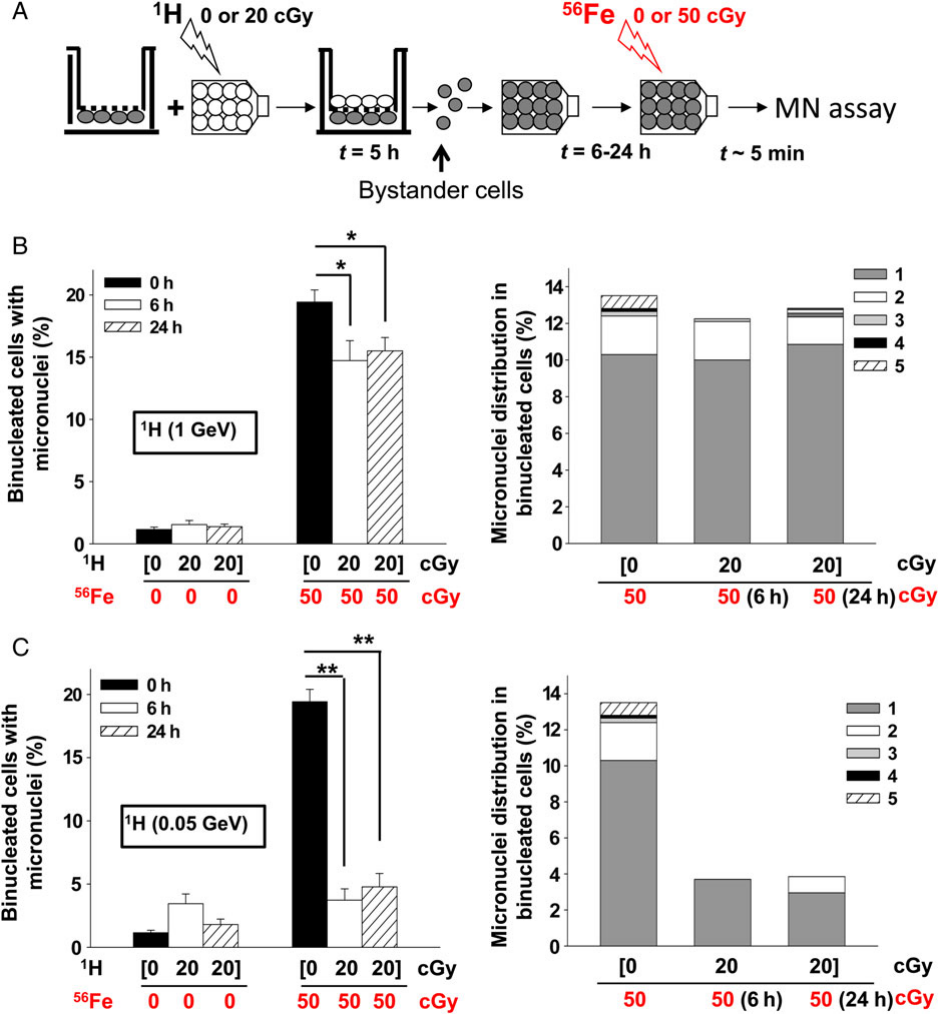
Proton-irradiation propagates protective bystander effects. (**A**) Experiment schematic: Proton-irradiated cells are plated on the top side of the porous membrane of a Transwell® insert, with bystander cells growing on its underside. Following ∼5 h of co-culture, the bystander cells were harvested and seeded in tissue culture flasks. The bystander cells were exposed to iron ions at different times after seeding. (**B**) Micronucleus formation (left panel) and micronuclei distribution (right panel) in bystander AG1522 cells that had been in co-culture with cells irradiated with 0 or 20 cGy (shown in brackets) from 1-GeV protons (^1^H). Bystander cells were exposed 0, 6 or 24 h later to 0 or 50 cGy from 1-GeV/u iron ions (^56^Fe). (**C**) Micronucleus formation (left panel) and micronuclei distribution (right panel) in bystander AG1522 cells that had been in co-culture with cells irradiated with 0 or 20 cGy (shown in brackets) from 0.05-GeV protons (^1^H). Bystander cells were exposed 0, 6 or 24 h later to 0 or 50 cGy from 1-GeV/u iron ions (^56^Fe). The data show that bystander cells that were co-cultured with low-dose proton-irradiated cells are protected from DNA damage induced by a subsequent challenge of energetic iron ions. *P < 0.05, **P < 0.0001.

Others have shown that protective effects induced by low doses of X-rays are propagated to unirradiated bystander cells [52]. To investigate the spread of adaptive responses from proton-irradiated cells to bystander cells in the vicinity, we co-cultured AG1522 cells exposed to 0 (sham) or 20 cGy of protons with bystander AG1522 cells using the layered tissue culture system (see scheme in Fig. 2A). Following a 5-h co-culture, the bystander cells were harvested with a high degree of purity (99.9%), as revealed by flow cytometry analyses of the transfer of CellTracker dye-loaded AG1522 cells cultured on the upper side of the membrane to cells growing on the underside of the membrane. The isolated bystander cells were seeded in tissue culture flasks at confluent density, and challenged 6, 24 or 72 h later with 50 cGy of 1 GeV/u iron ions (Fig. 2A). Within 5–10 min after the challenge dose, the cells were subcultured and submitted for assessment of micronucleus formation. When the bystander cells were challenged with iron ions within 24 h after isolation from the Transwell® inserts, a significant reduction in the fraction of micronucleated cells was observed (Fig. 2B). When the challenge dose was delivered at 6 h, 19.4 ± 1.0% of bystanders cells that were co-cultured with sham-treated cells were micronucleated vs 14.7 ± 1.6% when the bystanders were co-cultured with 1 GeV proton-irradiated cells (*n* = 2, *P* < 0.005) (Fig. 2B, left panel). A similar mitigating effect was observed at 24 h (19.4 ± 1.0 vs 15.5 ± 1.1%, *n* = 3, *P* < 0.005) (Fig. 2B, left panel). The priming proton dose also induced a decrease in the number of fragments in the micronucleated cells (Fig. 2B, right panel). However, the effect appears to be transient as the mitigating effect disappeared by 72 h (data not shown).

We have also observed protective bystander effects when AG1522 cells were exposed to 20 cGy of 0.05-GeV protons. A significant reduction in micronucleus formation was detected in bystander cells that were challenged with a mean absorbed dose of 50 cGy of 1 GeV/u iron ions (Fig. 2C). Relative to bystander cells co-cultured with sham-treated cells, the fraction of micronucleated cells was reduced from 19.4 ± 1.0 to 3.7 ± 0.9% at 6 h, and to 4.8 ± 1.1% at 24 h (*n* = 3, *P* < 0.0001) (Fig. 2C, left panel). As in the case of exposure to 1-GeV protons, the priming proton dose resulted in fewer micronucleated cells exhibiting more than three micronuclei (Fig. 2C, right panel).

Together, these data show that pre-exposure to a low dose of low-LET protons protects against DNA damage from a subsequent exposure to high-LET energetic iron particles (Fig. 1). Significantly, they indicate that the protective effects against high-LET iron ions are propagated to neighboring non-irradiated cells (Fig. 2). As measured by the endpoint of micronucleus formation, the effect appears to be transient, because it was undetectable when the challenge dose was delivered 72 h following the proton priming dose. Interestingly, our recent studies have shown that exposure of AG1522 cells to doses as low as 10 cGy of 1-GeV protons upregulates the level of the Translationally Controlled Tumor Protein (TCTP), which we have shown to be implicated in adaptive protection through its role in DNA damage sensing and repair [18].

Several studies have shown that high-LET particulate radiations (i.e. alpha and HZE) are potent inducers of harmful non-targeted effects when cell cultures are exposed to very low fluences of these particles. Further, several laboratories have examined the effect of sequential exposures to proton and iron ions on the yield of DNA damage [46, 50, 53, 54]. While these studies emphasized the need to investigate the interactions between targeted and non-targeted effects of radiation, they indicated that the outcome of sequential exposures to space radiation is complex. Whereas proton irradiation was shown to propagate stressful effects to bystander cells, pre-exposure of cells to protons protected them against clastogenic effects propagated via factors secreted by iron-ion-irradiated cells [53]. The results shown here indicate that exposure to a low dose of low-LET protons results in protective mechanisms that spare the targeted and the bystander cells in their vicinity from the harmful effects of a subsequent challenge by high-LET iron ions (Figs 1 and 2). For prolonged space travel, in which proton-induced non-targeted effects will occur and the resulting bystander cells will be traversed subsequently by HZE particles, the extension of ground-based mechanistic studies to investigate the effects of sequential doses to both low- and high-LET radiations delivered at low dose/low dose-rate may contribute to decision-making concerning radiation protection in space. These studies are also pertinent to the development of radiotherapy protocols using particulate radiations of different LET, and to the enhancement of our understanding of intercellular communication under oxidative stress conditions. Extending them to examine the cross-talk between biological responses induced by low-LET protons and high-LET HZE particles within intact tissues will improve understanding of the response of the entire biological system.

## FUNDING

This research is supported by NASA Grants NNJ06HD91G and NNX15AD62G. Funding to pay the Open Access publication charges for this article was provided by NASA grant NNX15AD62G.

## References

[1] Schimmerling W Space and radiation protection: scientific requirements for space research. Radiat Environ Biophys 1995;34:133–7. 7480626 10.1007/BF01211538

[2] Hada M Georgakilas AG Formation of clustered DNA damage after high-LET irradiation: a review. J Radiat Res 2008;49:203–10. 18413977 10.1269/jrr.07123

[3] Tobias CA Failla Memorial lecture. The future of heavy-ion science in biology and medicine. *Radiat Res* 1985;103:1–33. 3906741

[4] Zapp EN Townsend LW Cucinotta FA Solar particle event organ doses and dose equivalents for interplanetary crews: variations due to body size. Adv Space Res 2002;30:975–9. 12539772 10.1016/s0273-1177(02)00166-7

[5] Norbury JW Miller J Review of nuclear physics experimental data for space radiation. Health Phys 2012;103:640–2. 23032893 10.1097/HP.0b013e318261fb7f

[6] Cucinotta F Nikjoo H Goodhead DT The effect of delta rays on the number of particle traversals per cell in laboratory and space exposures. Radiat Res 1998;150:115–9. 9650608

[7] BEIR-VII. Health Risks from Exposure to Low Levels of Ionizing Radiation. Washington, DC: National Research Council of the National Academies, 2005. 25077203

[8] ICRP. 1990 Recommendations of the International Commission on Radiological Protection. ICRP Publication 60, Pergamon Press, Oxford, UK. Ann ICRP 1991;21 (1–3). 2053748

[9] Dauer LT Brooks AL Hoel DG Review and evaluation of updated research on the health effects associated with low-dose ionising radiation. Radiat Prot Dosimetry 2010;140:103–36. 20413418 10.1093/rpd/ncq141

[10] Li M Gonon G Buonanno M Health risks of space exploration: targeted and non-targeted oxidative injury by high-charge and high-energy particles. Antioxid Redox Signal 2014;20:1501–23. 24111926 10.1089/ars.2013.5649 PMC3936510

[11] Feinendegen LE Paretzke H Neumann RD Two principal considerations are needed after low doses of ionizing radiation. Radiat Res 2008;169:247–8. 18220467 10.1667/RR1123.1d

[12] Matsumoto H Hamada N Takahashi A Vanguards of paradigm shift in radiation biology: radiation-induced adaptive and bystander responses. J Radiat Res 2007;48:97–106. 17327685 10.1269/jrr.06090

[13] Mothersill C Seymour CB Radiation-induced bystander effects—implications for cancer. Nat Rev Cancer 2004;4:158–64. 14964312 10.1038/nrc1277

[14] Azzam EI de Toledo SM Little JB Stress signaling from irradiated to non-irradiated cells. Curr Cancer Drug Targets 2004;4:53–64. 14965267 10.2174/1568009043481641

[15] Hei TK Zhou H Chai Y Radiation induced non-targeted response: mechanism and potential clinical implications. Curr Mol Pharmacol 2011;4:96–105. 21143185 10.2174/1874467211104020096 PMC3356574

[16] Prise KM O'Sullivan JM Radiation-induced bystander signalling in cancer therapy. Nat Rev Cancer 2009;9:351–60. 19377507 10.1038/nrc2603 PMC2855954

[17] de Toledo SM Asaad N Venkatachalam P Adaptive responses to low-dose/low-dose-rate gamma rays in normal human fibroblasts: the role of growth architecture and oxidative metabolism. Radiat Res 2006;166:849–57. 17149977 10.1667/RR0640.1

[18] Zhang J de Toledo SM Pandey BN Role of the translationally controlled tumor protein in DNA damage sensing and repair. Proc Natl Acad Sci U S A 2012;109:E926–33. 22451927 10.1073/pnas.1106300109 PMC3341051

[19] Wolff S The adaptive response in radiobiology: evolving insights and implications. Environ Health Perspect 1998;106 Suppl 1:277–83. 9539019 10.1289/ehp.98106s1277 PMC1533272

[20] Kojima S Ishida H Takahashi M Elevation of glutathione induced by low-dose gamma rays and its involvement in increased natural killer activity. Radiat Res 2002;157:275–80. 11839089 10.1667/0033-7587(2002)157[0275:eogibl]2.0.co;2

[21] Wang B Ohyama H Nose T Adaptive response in embryogenesis: I. Dose and timing of radiation for reduction of prenatal death and congenital malformation during the late period of organogenesis. *Radiat Res* 1998;150:120–2. 9650609

[22] Cai L Liu SZ Induction of cytogenetic adaptive response of somatic and germ cells *in vivo* and *in vitro* by low-dose X-irradiation. Int J Radiat Biol 1990;58:187–94. 1973436 10.1080/09553009014551541

[23] Azzam EI Raaphorst GP Mitchel RE Radiation-induced adaptive response for protection against micronucleus formation and neoplastic transformation in C3H 10T1/2 mouse embryo cells. Radiat Res 1994;138:S28–31. 8146320

[24] Rigaud O Moustacchi E Radioadaptation to the mutagenic effect of ionizing radiation in human lymphoblasts: molecular analysis of HPRT mutants. Cancer Res 1994;54:1924s–8 s. 8137313

[25] Shadley JD Afzal V Wolff S Characterization of the adaptive response to ionizing radiation induced by low doses of X rays to human lymphocytes. Radiat Res 1987;111:511–7. 3659285

[26] Olivieri G Bodycote J Wolff S Adaptive response of human lymphocytes to low concentrations of radioactive thymidine. Science 1984;223:594–7. 6695170 10.1126/science.6695170

[27] Azzam EI de Toledo SM Raaphorst GP Low-dose ionizing radiation decreases the frequency of neoplastic transformation to a level below the spontaneous rate in C3H 10T1/2 cells. Radiat Res 1996;146:369–73. 8927708

[28] Redpath JL Antoniono RJ Induction of an adaptive response against spontaneous neoplastic transformation *in vitro* by low-dose gamma radiation. Radiat Res 1998;149:517–20. 9588363

[29] Baqai FP Gridley DS Slater JM Effects of spaceflight on innate immune function and antioxidant gene expression. J Appl Physiol 2009;106:1935–42. 19342437 10.1152/japplphysiol.91361.2008 PMC2692779

[30] Yang H Magpayo N Rusek A Effects of very low fluences of high-energy protons or iron ions on irradiated and bystander cells. Radiat Res 2011;176:695–705. 21988573 10.1667/rr2674.1

[31] Fournier C Barberet P Pouthier T No evidence for DNA and early cytogenetic damage in bystander cells after heavy-ion microirradiation at two facilities. Radiat Res 2009;171:530–40. 19580488 10.1667/RR1457.1

[32] Harada K Nonaka T Hamada N Heavy-ion-induced bystander killing of human lung cancer cells: role of gap junctional intercellular communication. Cancer Sci 2009;100:684–8. 19469013 10.1111/j.1349-7006.2009.01093.x PMC11159273

[33] Yang H Anzenberg V Held KD The time dependence of bystander responses induced by iron-ion radiation in normal human skin fibroblasts. Radiat Res 2007;168:292–8. 17705636 10.1667/RR0864.1

[34] National Research Council, Managing Space Radiation Risk in the New Era of Space Exploration. Washington, DC: The National Academies Press, 2008.

[35] Buonanno M de Toledo SM Azzam EI Increased frequency of spontaneous neoplastic transformation in progeny of bystander cells from cultures exposed to densely-ionizing radiation. PLoS One 2011;6:e21540. 21738697 10.1371/journal.pone.0021540 PMC3125249

[36] Buonanno M de Toledo SM Pain D Long-term consequences of radiation-induced bystander effects depend on radiation quality and dose and correlate with oxidative stress. Radiat Res 2011;175:405–15. 21319986 10.1667/RR2461.1 PMC3106980

[37] Gonon G Groetz JE de Toledo SM Nontargeted stressful effects in normal human fibroblast cultures exposed to low fluences of high charge, high energy (HZE) particles: kinetics of biologic responses and significance of secondary radiations. Radiat Res 2013;179:444–57. 23465079 10.1667/RR3017.1 PMC3995407

[38] Autsavapromporn N Plante I Liu C Genetic changes in progeny of bystander human fibroblasts after microbeam irradiation with X-rays, protons or carbon ions: the relevance to cancer risk. Int J Radiat Biol 2015;91:62–70. 25084840 10.3109/09553002.2014.950715

[39] Venkatachalam P de Toledo SM Pandey BN Regulation of normal cell cycle progression by flavin-containing oxidases. Oncogene 2008;27:20–31. 17637756 10.1038/sj.onc.1210634

[40] Terasima T Tolmach LJ Changes in x-ray sensitivity of HeLa cells during the division cycle. Nature 1961;190:1210–1. 13775960 10.1038/1901210a0

[41] Gray LH Conger AD Ebert M The concentration of oxygen dissolved in tissues at the time of irradiation as a factor in radiotherapy. Br J Radiol 1953;26:638–48. 13106296 10.1259/0007-1285-26-312-638

[42] Rueckert RR Mueller GC Effect of oxygen tension on HeLa cell growth. Cancer Res 1960;20:944–9. 14440030

[43] Autsavapromporn N de Toledo SM Jay-Gerin JP Human cell responses to ionizing radiation are differentially affected by the expressed connexins. J Radiat Res 2013;54:251–9. 23139176 10.1093/jrr/rrs099 PMC3589937

[44] Zhao Y De Toledo SM Hu G Connexins and cyclooxygenase-2 crosstalk in the expression of radiation-induced bystander effects. Br J Cancer 2014;111:125–31. 24867691 10.1038/bjc.2014.276 PMC4090739

[45] Fenech M Morley AA Measurement of micronuclei in lymphocytes. Mutat Res 1985;147:29–36. 3974610 10.1016/0165-1161(85)90015-9

[46] Sutherland BM Cuomo NC Bennett PV Induction of anchorage-independent growth in primary human cells exposed to protons or HZE ions separately or in dual exposures. Radiat Res 2005;164:493–6. 16187755 10.1667/rr3357.1

[47] Zhou G Bennett PV Cutter NC Proton-HZE-particle sequential dual-beam exposures increase anchorage-independent growth frequencies in primary human fibroblasts. Radiat Res 2006;166:488–94. 16953667 10.1667/RR0596.1

[48] Bennett PV Cutter NC Sutherland BM Split-dose exposures versus dual ion exposure in human cell neoplastic transformation. Radiat Environ Biophys 2007;46:119–23. 17256176 10.1007/s00411-006-0091-y

[49] Hada M Meador JA Cucinotta FA Chromosome aberrations induced by dual exposure of protons and iron ions. Radiat Environ Biophys 2007;46:125–9. 17237947 10.1007/s00411-006-0083-y

[50] Elmore E Lao XY Kapadia R Neoplastic transformation *in vitro* by mixed beams of high-energy iron ions and protons. Radiat Res 2011;176:291–302. 21732791 10.1667/rr2646.1

[51] Azzam EI Jay-Gerin JP Pain D Ionizing radiation-induced metabolic oxidative stress and prolonged cell injury. Cancer Lett 2012;327:48–60. 22182453 10.1016/j.canlet.2011.12.012 PMC3980444

[52] Klammer H Kadhim M Iliakis G Evidence of an adaptive response targeting DNA nonhomologous end joining and its transmission to bystander cells. Cancer Res 2010;70:8498–506. 20861183 10.1158/0008-5472.CAN-10-1181

[53] Yang H Magpayo N Held KD Targeted and non-targeted effects from combinations of low doses of energetic protons and iron ions in human fibroblasts. Int J Radiat Biol 2011;87:311–9. 21158498 10.3109/09553002.2010.537431

[54] Hada M Cucinotta FA Gonda SR mBAND analysis of chromosomal aberrations in human epithelial cells exposed to low- and high-LET radiation. Radiat Res 2007;168:98–105. 17722995 10.1667/RR0759.1

